# Draft genomes of *Shigella* strains used by the STOPENTERICS consortium

**DOI:** 10.1186/s13099-015-0061-5

**Published:** 2015-06-04

**Authors:** Omar Rossi, Kate S Baker, Armelle Phalipon, François-Xavier Weill, Francesco Citiulo, Philippe Sansonetti, Christiane Gerke, Nicholas R Thomson

**Affiliations:** Novartis Vaccines Institute for Global Health, s.r.l., a GSK Company, Siena, Italy; Wellcome Trust Sanger Institute, Hinxton, UK; Institut Pasteur, Paris, France

**Keywords:** *Shigella*, STOPENTERICS, Genome, Vaccine

## Abstract

**Background:**

Despite a significant global burden of disease, there is still no vaccine against shigellosis widely available. One aim of the European Union funded STOPENTERICS consortium is to develop vaccine candidates against *Shigella.* Given the importance of translational vaccine coverage, here we aimed to characterise the *Shigella* strains being used by the consortium by whole genome sequencing, and report on the stability of strains cultured in different laboratories or through serial passage.

**Methods:**

We sequenced, de novo assembled and annotated 20 *Shigella* strains being used by the consortium. These comprised 16 different isolates belonging to 7 serotypes, and 4 derivative strains. Derivative strains from common isolates were manipulated in different laboratories or had undergone multiple passages in the same laboratory. Strains were mapped against reference genomes to detect SNP variation and phylogenetic analysis was performed.

**Results:**

The genomes assembled into similar total lengths (range 4.14–4.83 Mbp) and had similar numbers of predicted coding sequences (average of 4,400). Mapping analysis showed the genetic stability of strains through serial passages and culturing in different laboratories, as well as varying levels of similarity to published reference genomes. Phylogenetic analysis revealed the presence of three main clades among the strains and published references, one containing the *Shigella flexneri* serotype 6 strains, a second containing the remaining *S. flexneri* serotypes and a third comprised of *Shigella sonnei* strains.

**Conclusions:**

This work increases the number of the publically available *Shigella* genomes available and specifically provides information on strains being used for vaccine development by STOPENTERICS. It also provides information on the variability among strains maintained in different laboratories and through serial passage. This work will guide the selection of strains for further vaccine development.

## Background

*Shigella* are Gram-negative bacteria that represent the etiologic agent of the shigellosis, a global human health problem, especially in developing countries and in children younger than 5 years. Shigellosis is estimated to cause annually 125 million cases and 100,000 deaths [1], and is one of main causes of traveller’s diarrhea. The genus *Shigella* comprises four serogroups (*Shigella dysenteriae*, *Shigella sonnei*, *Shigella flexneri* and *Shigella boydii*) subdivided in 50 different serotypes based on the carbohydrate composition of the O antigen of their lipopolysaccharide [2] and the presence of serotypes varies among different regions and over time [3]. As no vaccines are currently widely available, one of the aims of the European Union-funded STOPENTERICS consortium (Vaccination against *Shigella* and ETEC: novel antigens, novel approaches) [4] is to develop novel vaccine candidates against *Shigella* [e.g. the Generalized Modules for Membrane Antigens (GMMA) approach [5, 6]], as well as to improve the immunogenicity of the existing antigens (e.g. synthetic chemistry for glycoconjugates [7]). To this end, partners of the STOPENTERICS consortium have been integrating basic research, particularly genomics, transcriptomics, proteomics, and other high-throughput technologies, with novel vaccine technologies and synthetic chemistry [7]. To assemble *Shigella* expertise to identify and rapidly take novel vaccine candidates through to clinical trials for effective vaccine development, the research is carried out among different academic institutions (e.g. University of Oxford, Wellcome Trust Sanger Institute, Institut Pasteur) and vaccines companies (Novartis Vaccines Institute for Global Health and Sanofi-Pasteur).

To ensure the congruence of strains between laboratories, and create a public resource for vaccine development and further *Shigella* research, we whole genome sequenced the *Shigella* strains used by the STOPENTERICS consortium which are used as they offer most effective breadth of cross-protection against *Shigella* sp. in endemic areas [8], and report the assembly and annotation of their draft genomes. We assessed the presence of SNPs between strains and against references, as well as defined their phylogenetic relationships, and compared genetic stability of strains maintained in different consortium laboratories and after serial passage.

## Methods

### Bacterial strains

The *Shigella* strains analysed in this study and relevant metadata are summarized in Table 1. Strains were serotyped by slide agglutination using commercially available monovalent antisera (Denka Seiken, Japan) to all type specific somatic antigens and the group factor antigens [9].

### DNA extraction and genome sequencing

Bacterial cultures were grown over night in liquid Luria–Bertani (LB) media to an optical density (measured at 600 nm) of approximately three. Genomic DNA was isolated using the Wizard kit (Promega, Madison, WI, USA) according to manufacturer’s instructions. Purified DNA was then sequenced at the Wellcome Trust Sanger Institute (WTSI). Paired end libraries 150 bp in length were generated and sequenced on the Illumina MiSeq instrument (San Diego, CA, USA) according to in house protocols [10, 11], with an approximately 500 bp insert size. Sequence data for each of the strains were deposited in the European Nucleotide Archive (accession numbers in Table 1).

### Genomic analysis

Resulting sequencing reads were trimmed using Trimmomatic v0.27 [12] to remove adapters, bases with a PHRED score of <30, and remaining reads with lengths <50 bp.

High quality reads were then mapped to relevant reference strains (Table 1), using SMALT (http://www.sanger.ac.uk/resources/software/smalt/) and Single Nucleotide Polymorphisms (SNPs) were called using Samtools [13]. Nucleotides where mapping quality was below 30 and genotyping quality was below 50 were excluded from further analysis. Mapping coverage of all isolates was approximately 70-fold coverage.

De novo assembly was performed using Velvet Optimiser [14] and contiguous sequences were annotated using Prokka [15]. Clustering and BLAST comparisons were used to determine the presence/absence of genes in annotated assemblies as previously described [16].

To prepare a multiple sequence alignment for phylogenetic analysis, sequencing data from strains in this study and from simulated fastq data created from published reference genomes were mapped to the chromosome of *S. flexneri* 2457T (GenBank accession: NC_004741.1). The other reference isolates (and their accessions) used in this analysis were: *S. sonnei* Ss046 (NC_007384.1), *S. sonnei* 53G (NC_016822.1), *S. flexneri* 5 M90T (AGNM01000000), *S. flexneri* 5a 8401 (NC_008258.1), *S. flexneri* 2a NCTC1 (LM651928), *S. flexneri* 2a 301 (NC_004337.2), *S. flexneri* X 2002017 (NC_017328.1) and *S. boydii* Sb 227 (NC_007613.1). Core genes (n = 2,427) were identified that had 100% mapping coverage in all isolates and phylogenetic analysis was performed using RAxML software v7.0.3 [17] on the 43,349 variable sites (subset from 2,306,256 bp) of these core genes.

In silico molecular serotyping of *S. flexneri* isolates was performed on de novo assemblies for each isolate (and as in [18]). Briefly, the presence/absence and known differences of the *gtr* genes (encoding for enzymes responsible of the presence of type specific antigens I, II, IV, V, X, IC), *oac* genes (encoding for enzymes that mediates O-acetylation modification in serotypes 1b, 3a, 3b, and 4b) and *wzx*6 (specific for serotype 6) were analyzed, facilitating the differentiation of the six different *S. flexneri* serotypes.

## Results and discussion

Sixteen different *Shigella* isolates belonging to seven different serotypes were sequenced (listed in Table 1). These included *S. sonnei* (2 isolates) and different *S. flexneri* serotypes including 1a, 1b (2 isolates), 2a, 3a, 5a and 6 (eight different isolates) plus four derivative strains from either serial passage (*S. sonnei* 53G, *S. flexneri* 2a 2457T) or having been cultivated and the DNA extracted in different laboratories (*S.* *flexneri* 3a 6865 and *S. flexneri* 6 10.5302). Derivative strains from the same isolate, but manipulated in different laboratories of the STOPENTERICS consortium were denoted ‘_1’ and ‘_2’, whereas those that had undergone serial passage (~10 passages) in the same laboratory were denoted ‘_p’. The derivatives allowed us to assess the genetic stability of strains across laboratories and through serial passage.

**Table 1:** Summary results assembly, annotation and mapping

Name in the study	True name, country of infection, year of isolation	Sample run accession	Sample accession	De novo assembly genomic size	Contigs number	Average Contigs Length	N50	CDS detected	Reference used for mapping	Number of SNPs detected	% of reference mapped
Ss_53G	Korea, 2000	ERS387232	ERR477376	4,698,814	402	11,688.59	29,856	4,495	Ss 53G	2	92.86
Ss_53G_p	Korea, 2000	ERS387243	ERR477387	4,832,559	406	11,902.85	28,177	4,655	Ss 53G	2	92.80
Ss_25931	Unknown	ERS387235	ERR477379	4,799,852	426	11,267.26	27,765	4,578	Ss 53G	630	89.51
Sf 1a_Sh07.3008	Sh07-3008, Cameroon, 2007	ERS445026	ERR573382	4,139,080	265	15,619.17	34,771	4,044	Sf 2a 2457T	3,459	86.58
Sf 1b_Sh04.7434	Sh04-7432, Tunisia, 2004	ERS445024	ERR573380	4,402,078	314	14,019.36	34,552	4,342	Sf 2a 2457T	2,935	87.63
Sf 1b_Sh04.9462	Sh04-9462, Cameroon, 2004	ERS445025	ERR573381	4,272,358	280	15,258.42	34,756	4,206	Sf 2a 2457T	3,207	87.36
Sf 2a_2457T	Japan, 1954	ERS387233	ERR477377	4,681,429	344	13,608.81	35,441	4,583	Sf 2a 2457T	195	93.72
Sf 2a_2457T_p	Japan, 1954	ERS387242	ERR477386	4,697,211	343	13,694.49	35,151	4,605	Sf 2a 2457T	192	93.88
Sf 3a_6865_1	Unknown	ERS387236	ERR477380	4,665,099	335	13,925.67	35,495	4,550	Sf 2a 2457T	7,543	86.79
Sf 3a_6865_2	Unknown	ERS445023	ERR573379	4,704,030	330	14,254.64	35,991	4,580	Sf 2a 2457T	7,708	87.08
Sf 5a_M90T	Unknown <1980	ERS387234	ERR477378	4,486,899	327	13,721.40	32,160	4,391	Sf 5a M90T	25	97.82
Sf 6_Sh10.5302_1	201005302, Madagascar, 2010	ERS387237	ERR477381	4,414,146	446	9,897.19	22,838	4,269	Sb Sb227	4,408	89.30
Sf 6_Sh10.5302_2	201005302, Madagascar, 2010	ERS445029	ERR573385	4,351,336	426	10,214.4	22,774	4,168	Sb Sb227	4,406	89.10
Sf 6_Sh10.3933	201003933, Nigeria, 2010	ERS387238	ERR477382	4,508,368	433	10,411.94	23,090	4,386	Sb Sb227	4,456	89.33
Sf 6_Sh10.8537	201008537, Egypt, 2010	ERS387239	ERR477383	4,524,547	425	10,645.99	23,238	4,398	Sb Sb227	4,451	89.77
Sf 6_Sh10.6306	201006306, India, 2010	ERS387240	ERR477384	4,481,178	439	10,207.69	23,066	4,367	Sb Sb227	4,467	89.44
Sf 6_Sh10.6237	201006237, Mexico, 2010	ERS387241	ERR477385	4,528,968	434	10,435.41	24,012	4,397	Sb Sb227	4,389	89.39
Sf 6_NCDC.2924-71	NCDC 2924-71, Unknown, 1971	ERS445027	ERR573383	4,392,208	413	10,634.89	22,784	4,246	Sb Sb227	4,288	89.66
Sf 6_Sc544	Unknown, <1977	ERS445028	ERR573384	4,430,667	415	10,676.31	22,494	4,302	Sb Sb227	4,296	88.82
Sf 6_Sh11.10088	201110088, France (Reunion Island), 2011	ERS445030	ERR573386	4,547,256	423	10,750.01	23,991	4,428	Sb Sb227	4,483	89.88

Results of genomic assembly and annotation were similar for all strains (Table 1). The strains assembled into an average of 381 contigs (range 265–446), with an average contigs length of 12,141 bp (range 9,897–15,619) and an N50 of 28,620 (range 22,494–35,991). The resulting genomic size was similar for all the strains and fell within the range of 4.14–4.83 Mbp. Similarly, automated annotation predicted the presence of an average of 4,400 coding sequences per genome (range 4,044–4,583; Table 1). The serotypes of the *Shigella* strains were confirmed based on the combinations of *gtr* and *oac* genes, encoding the relevant enzymes for the serotype-specific OAg modifications [18] (not shown).

To facilitate strain comparisons and phylogenetic analysis, sequence reads were mapped to existing *Shigella* reference genomes (Table 1). The percentage of the reference genome covered by mapped reads ranged from 87 to 98% and the number of SNPs varied (Table 1) depending on the isolate. These data showed comparatively few SNPs (<200) when an isolate was compared to a previously published reference of itself (as in the case of *S. sonnei* 53G, *S. flexneri* 2a 2457T, *S.* *flexneri* 5a M90T). Higher numbers of SNPs were seen where no such reference was available. For example, when an isolate was mapped to a reference genome of a different isolate of the same serotype (e.g. Ss_25931 mapped against Ss_53G) several hundred SNPs were seen, and several thousand SNPs were seen if the isolate was mapped to a reference isolate from a phylogenetic related, but distinct serotype (e.g. *S. flexneri* six isolates mapped against *S. boydii* strain Sb227).

To assess the genomic stability of isolates held at different laboratories and through serial passage within the same laboratory, we resequenced a number of isolates and compared their mapping results to the relevant reference (Table 1). Two isolates (original and passaged) of *S. sonnei* 53G had only two SNPs relative to the published reference genome, and these SNPs were the same in both isolates. Similarly, the sequences of original and passaged *S. flexneri* 2a strain 2457T were very similar, but had 195 and 192 SNPs relative to the published reference genome. Among these SNPs, 188 were common to both isolates and the remaining four and seven sites were not resolved in the other isolate, indicating that the two isolates were likely identical to each other. The level of genetic variation compared to the reference strain was surprising (~200 SNPs) and may have biological significance, showing the utility of obtaining up-to-date genetic information for the exact strain being worked with in a given project. Two strains, Sf 3a_6865 and Sf 6_10.5302, were manipulated for sequencing in separate laboratories in the consortium. These strains differed by only one and two SNPs respectively, indicating that over a 2–3 year time period, isolate genomes remain relatively stable through passage and between laboratories, but may differ significantly from published references.

**Figure 1:**
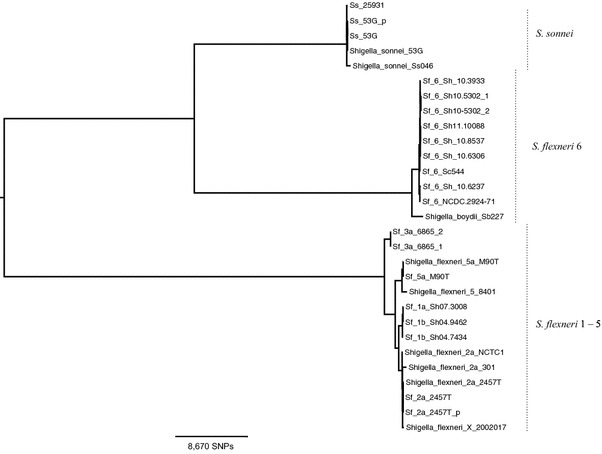
Mid-point rooted maximum likelihood phylogeny of strains based on core genome. Names of strains sequenced in this study are abbreviated and those of reference genomes are given in full.

To assess the phylogenetic relationship of the isolates, we constructed a maximum likelihood phylogenetic tree of a large core genome shared among the strains (Figure 1). Consistent with expectations based on prior evolutionary studies of shigellae [19, 20], the strains were divided into three main clades, with the *S. flexneri* six strains being phylogenetically removed from the remaining *S. flexneri* serotypes, and the *S. sonnei* strains forming a separate clade.

## Conclusions

The work presented here increases the number of publically available *Shigella* genomes, including for the first time, sequencing data for *S. sonnei* 25931, two *S. flexneri* 1b, one *S. flexneri* 1a, one *S. flexneri* 3a and 8 *S. flexneri* six isolates. We provide details on the draft genomes generated from this sequencing data, and report SNP variation in strains maintained in different laboratories and after serial passage. We also described the relatedness of the strains and isolates used by the STOPENTERICS consortium, and have deposited this data as a public resource. Data presented in this work will guide the selection of strains for further development of vaccine and contribute to a growing awareness of diversity in *Shigella*.

## Abbreviations

SNP: single nucleotide polymorphism
Ss: *Shigella sonnei*
Sf: *Shigella flexneri*
Sb: *Shigella boydii*

## References

[1] Lozano R, Naghavi M, Foreman K, Lim S, Shibuya K, Aboyans V (2012). Global and regional mortality from 235 causes of death for 20 age groups in 1990 and 2010: a systematic analysis for the Global Burden of Disease Study 2010. Lancet.

[2] Liu B, Knirel YA, Feng L, Perepelov AV, Senchenkova SN, Wang Q (2008). Structure and genetics of *Shigella* O antigens. FEMS Microbiol Rev.

[3] Levine MM, Kotloff KL, Barry EM, Pasetti MF, Sztein MB (2007). Clinical trials of *Shigella* vaccines: two steps forward and one step back on a long, hard road. Nat Rev Microbiol.

[4] STOPENTERICS, FP7/2007-2013, http://stopenterics.bio-med.ch/cms/default.aspx. Accessed 22 Apr 2015

[5] Berlanda Scorza F, Colucci AM, Maggiore L, Sanzone S, Rossi O, Ferlenghi I (2012). High yield production process for *Shigella* outer membrane particles. PLoS One.

[6] Rossi O, Pesce I, Giannelli C, Aprea S, Caboni M, Citiulo F (2014). Modulation of endotoxicity of *Shigella* generalized modules for membrane antigens (GMMA) by genetic lipid A modifications: relative activation of TLR4 and TLR2 pathways in different mutants. J Biol Chem.

[7] Gauthier C, Chassagne P, Theillet FX, Guerreiro C, Thouron F, Nato F (2014). Non-stoichiometric O-acetylation of *Shigella**flexneri* 2a O-specific polysaccharide: synthesis and antigenicity. Org Biomol Chem.

[8] Livio S, Strockbine N, Panchalingam S, Tennant SM, Barry EM, Marohn ME (2014). *Shigella* isolates from the Global Enteric Multicenter Study (GEMS) Inform Vaccine Development. Clin Infect Dis.

[9] Carlin NI, Lindberg AA (1986). Monoclonal antibodies specific for *Shigella flexneri* lipopolysaccharides: clones binding to type I and type III: 6, 7, 8 antigens, group 6 antigen, and a core epitope. Infect Immun.

[10] Quail MA, Kozarewa I, Smith F, Scally A, Stephens PJ, Durbin R (2008). A large genome center’s improvements to the Illumina sequencing system. Nat Methods.

[11] Quail MA, Otto TD, Gu Y, Harris SR, Skelly TF, McQuillan JA (2012). Optimal enzymes for amplifying sequencing libraries. Nat Methods.

[12] Bolger AM, Lohse M, Usadel B (2014). Trimmomatic: a flexible trimmer for Illumina sequence data. Bioinformatics.

[13] Li H, Handsaker B, Wysoker A, Fennell T, Ruan J, Homer N (2009). The sequence alignment/map format and SAMtools. Bioinformatics.

[14] Zerbino DR (2010). Using the Velvet de novo assembler for short-read sequencing technologies. Curr Protoc Bioinform.

[15] Seemann T (2014). Prokka: rapid prokaryotic genome annotation. Bioinformatics.

[16] Baker KS, Mather AE, McGregor H, Coupland P, Langridge GC, Day M (2014). The extant World War 1 dysentery bacillus NCTC1: a genomic analysis. Lancet.

[17] Stamatakis A (2006). RAxML-VI-HPC: maximum likelihood-based phylogenetic analyses with thousands of taxa and mixed models. Bioinformatics.

[18] Ashton PM, Baker KS, Gentle A, Wooldridge DJ, Thomson NR, Dallman TJ (2014). Draft genome sequences of the type strains of *Shigella flexneri* held at Public Health England: comparison of classical phenotypic and novel molecular assays with whole genome sequence. Gut Pathog.

[19] Pupo GM, Lan R, Reeves PR (2000). Multiple independent origins of *Shigella* clones of *Escherichia coli* and convergent evolution of many of their characteristics. Proc Natl Acad Sci USA.

[20] Yang J, Nie H, Chen L, Zhang X, Yang F, Xu X (2007). Revisiting the molecular evolutionary history of *Shigella* spp. J Mol Evol.

